# Using synthetic dataset for semantic segmentation of the human body in the problem of extracting anthropometric data

**DOI:** 10.3389/frai.2024.1336320

**Published:** 2024-08-09

**Authors:** Azat Absadyk, Olzhas Turar, Darkhan Akhmed-Zaki

**Affiliations:** Department of Science and Innovation, Astana IT University, Astana, Kazakhstan

**Keywords:** synthetic data, human segmentation, anthropometry, CNN, NVIDIA replicator, human body

## Abstract

**Background:**

The COVID-19 pandemic highlighted the need for accurate virtual sizing in e-commerce to reduce returns and waste. Existing methods for extracting anthropometric data from images have limitations. This study aims to develop a semantic segmentation model trained on synthetic data that can accurately determine body shape from real images, accounting for clothing.

**Methods:**

A synthetic dataset of over 22,000 images was created using NVIDIA Omniverse Replicator, featuring human models in various poses, clothing, and environments. Popular CNN architectures (U-Net, SegNet, DeepLabV3, PSPNet) with different backbones were trained on this dataset for semantic segmentation. Models were evaluated on accuracy, precision, recall, and IoU metrics. The best performing model was tested on real human subjects and compared to actual measurements.

**Results:**

U-Net with EfficientNet backbone showed the best performance, with 99.83% training accuracy and 0.977 IoU score. When tested on real images, it accurately segmented body shape while accounting for clothing. Comparison with actual measurements on 9 subjects showed average deviations of −0.24 cm for neck, −0.1 cm for shoulder, 1.15 cm for chest, −0.22 cm for thallium, and 0.17 cm for hip measurements.

**Discussion:**

The synthetic dataset and trained models enable accurate extraction of anthropometric data from real images while accounting for clothing. This approach has significant potential for improving virtual fitting and reducing returns in e-commerce. Future work will focus on refining the algorithm, particularly for thallium and hip measurements which showed higher variability.

## 1 Introduction

The extraction of anthropometric data has become relevant recently. This surge in importance is not solely driven by consumer desire for suitably sized products upon delivery but also by commercial interests in mitigating the wastage of apparel merchandise. Ignorance concerning one's bodily dimensions leads to challenges in clothing selection, thereby increasing the propensity for errors. Standard labels of clothing sizes are not always the same for various brands (looksize.com, 2020). For example, the M size of one brand may differ from the M of another brand, necessitating resources such as online size charts that detail the specifications for numerous brands. Clothes that are incorrectly sized are returned to the seller, thereby escalating logistical and warehousing costs associated with the returned merchandise. The returned items do not go back to store shelves but are thrown into a landfill in most cases (qz.com, 2016), it can be also recycled though it requires also more logistics hence the higher associated cost.

The convenience of online shopping and the availability of free return policies have resulted in a rise in e-commerce returns. While many returned items are still in excellent condition, they cannot be resold due to damage incurred during packaging and handling. As a result, these items often end up in landfills or are incinerated. This harms the environment, as it involves using resources for manufacturing, transportation, and disposal of the products (Igini, 2023). If consumers had precise knowledge of their body measurements, it would greatly diminish the need for returns and subsequently mitigate the environmental impact associated with them.

Existing neural networks for determining anthropometric data have different limitations, which complicate their application. We have created a straightforward algorithm that utilizes two architectures to calculate body measurements. This algorithm combines a CNN (Convolutional Neural Network) model for semantic segmentation and a model for human body posture analysis. However, when identifying specific body points through semantic segmentation, the algorithm doesn't consider the person's clothing, which complicates obtaining accurate data. Additionally, the available training datasets also have a shortage of information regarding clothing and only focus on determining body contours along with the garments.

The project's objectives include the construction of a semantic segmentation architecture trained on synthetic data that can be applied to real-world data, which is the main goal of this paper. Therefore, Section 4.1 describes the creation of a synthetic dataset that accurately represents a person's clothing while determining the actual body silhouette. Furthermore, Section 4.2 details the training of existing neural networks using the newly developed dataset. In Section 5, the created architectures are tested using real images, and the outcomes are compared with architectures trained on pre-existing data.

## 2 Related works

In recent years, there has been a growing interest in developing accurate and efficient methods for extracting anthropometric measurements from 3D scans and images. Many of these methods rely on deep learning architectures, which have shown promising results in accuracy and robustness. The proposed methods vary in terms of input data types, such as 2D images, 3D point clouds, and binary silhouettes, and the use of landmark detection or template fitting algorithms.

Most of the methods use digital image processing, convolutional neural networks, and machine learning methods to calculate body size from images (Lin and Wang, 2011; Jiang et al., 2012; de Souza et al., 2020). A parametric model of the body is also used to estimate shape parameters from binary silhouettes or shaded images (Dibra et al., 2016). Additionally, some also used images from a dataset of more than 4,000 people (Yan and Kämäräinen, 2021). Meanwhile, there is also a mobile automatic system for measuring the human body (Xia et al., 2018). Furthermore, some approaches evaluate the three-dimensional shape of the human body from two-dimensional images of the silhouette or images of a person in clothes, using a database of human body shapes (Chang and Wang, 2015; Song et al., 2016; Shigeki et al., 2018; Ji et al., 2019) or geodetic surface paths, using CNN's deep architecture as a basic method (Yan et al., 2021). Another paper proposes formula-driven supervised learning (FDSL), a method that automatically generates image patterns and their category labels using mathematical formulas, such as fractals, to create large-scale labeled datasets for pre-training convolutional neural networks without relying on natural images (Kataoka et al., 2021).

Several methods use a 3D model and are created using 3D scanners with a marker (Xiaohui et al., 2018), without body markers using mathematical definitions and image processing methods (Leong et al., 2007), or from a single point cloud structured using a grid (Škorvánková et al., 2022). For example, one proposes a deep neural network architecture that can accurately extract three-dimensional anthropometric measurements from frontal scans (Kaashki et al., 2023), a similar approach to AM-DL, which uses deep learning (Kaashki et al., 2021).

Several papers suggest new methods for creating synthetic data, such as using Unity Perception to create annotated datasets (Borkman et al., 2021). Other articles present synthetic datasets such as SURREAL (Varol et al., 2017), which includes 6 million images of people created from motion capture data, and Synscapes (Wrenninge and Unger, 2018), a dataset for analyzing street scenes created using photorealistic imaging techniques, or even a synthetic data generators for human-centered vision (Ebadi et al., 2021, 2022).

Most of the developed architectures work in specially created environments, such as a uniform background, tight clothing of a person (Leong et al., 2007; Jiang et al., 2012), and are designed to produce a degree of obesity that has not even obtained the desired results (Affuso et al., 2018). The results of the work can only be used in special conditions. Some use a three-dimensional body scanner to obtain a three-dimensional model with further processing in a neural network (Xiaohui et al., 2018; Kaashki et al., 2021; Škorvánková et al., 2022). These methods complicate their application in everyday life since not everyone has three-dimensional scanners. The application of anthropometric data detection technology was intended to be simplified and made more accessible. Our method combines the results of the two architectures to extract body dimensions. It makes it possible to determine the characteristic points of the neck, chest, waist, hip, and shoulder width from both the image and the body.

Existing datasets for semantic human segmentation also do not consider clothing (Wrenninge and Unger, 2018; Ebadi et al., 2021). Datasets such as Cityscapes (Cordts et al., 2016), ADE20K (Zhou et al., 2016), Syncscapes (Wrenninge and Unger, 2018) are mainly designed for street scene analysis, and COCO (Lin et al., 2014), PASCAL VOC (Everingham et al., 2009) are used for object detection and segmentation of several dozen classes in addition to humans. There are also synthetic datasets for Human-Centric Computer Vision Models, such as SURREAL (Varol et al., 2017) and PeopleSansPeople (Ebadi et al., 2021). And there is dataset from the social network TikTok to determine the silhouette of a dancing person from a image (Roman, 2023). In the task of defining anthropometric data, you can use these datasets. But all these datasets, with semantic segmentation, take into account a person's clothes. It is known that when measuring the size of the human body, clothing is not considered. And for this, we need a dataset that will take into account a person's clothing when determining the silhouette. Given the complexity of creating such a set, it was decided to create a synthetic dataset using NVIDIA Omniverse Replicator.

## 3 Problem statement

Given an input image X of size H x W (height x width), where each pixel location (i, j) has a set of features x(i, j) (RGB color values), need to find:


$$\hat{Y}=P\left(X,Y\right)$$

where *X* = {*x*(*i, j*)| *x*(*i, j*)∈[0, 255] *for all* 3 *channels*}−input image, *Y* = {*y*(*i, j*)| *y*(*i, j*)∈{0, 1}}−ground truth mask (1 - human body, 0 - background),Ŷ = {ŷ(*i, j*)| ŷ(*i, j*)∈{0, 1}}−predicted mask (1 - human body, 0 - background).

The function *P* is represented by a convolutional neural network (CNN) and is trained by minimizing a loss function L that measures the discrepancy between the predicted and true segmentation masks. We have used 2 types of loss functions. First is the binary cross-entropy loss function summarized on per-pixel basis:


$$L\;=\;\frac{1}{H*W}\sum_{i=0}^{H}\sum_{j=0}^{W}l(y(i,j),\;\hat{y}(i,\;j)),$$

where:


l(ξ, η)=− (ξ ∗ log(η) − (1−ξ) ∗ log(1−η)), ξ,η∈{0,1}

and the second is IoU loss function:


$$L(Y,\;\hat{Y})=1-\;IoU\left(Y,\;\hat{Y}\right)$$

where:


$$IoU\left(Y,\;\hat{Y}\right)\;=\;\left|Y\cap \hat{Y}\right|/\left|Y\cup \hat{Y}+\varepsilon \right|$$

where ||−represents the cardinality (i.e., count of pixels in the case of binary image masks), ∩− represents intersection, ∪−represents union, ε−a small positive constant such as 1e-15 to ensure numerical stability.

The model is trained to minimize this loss, which effectively maximizes the similarity of the outputs to the ground truth labels.

## 4 Materials and methods

**Figure 1:**
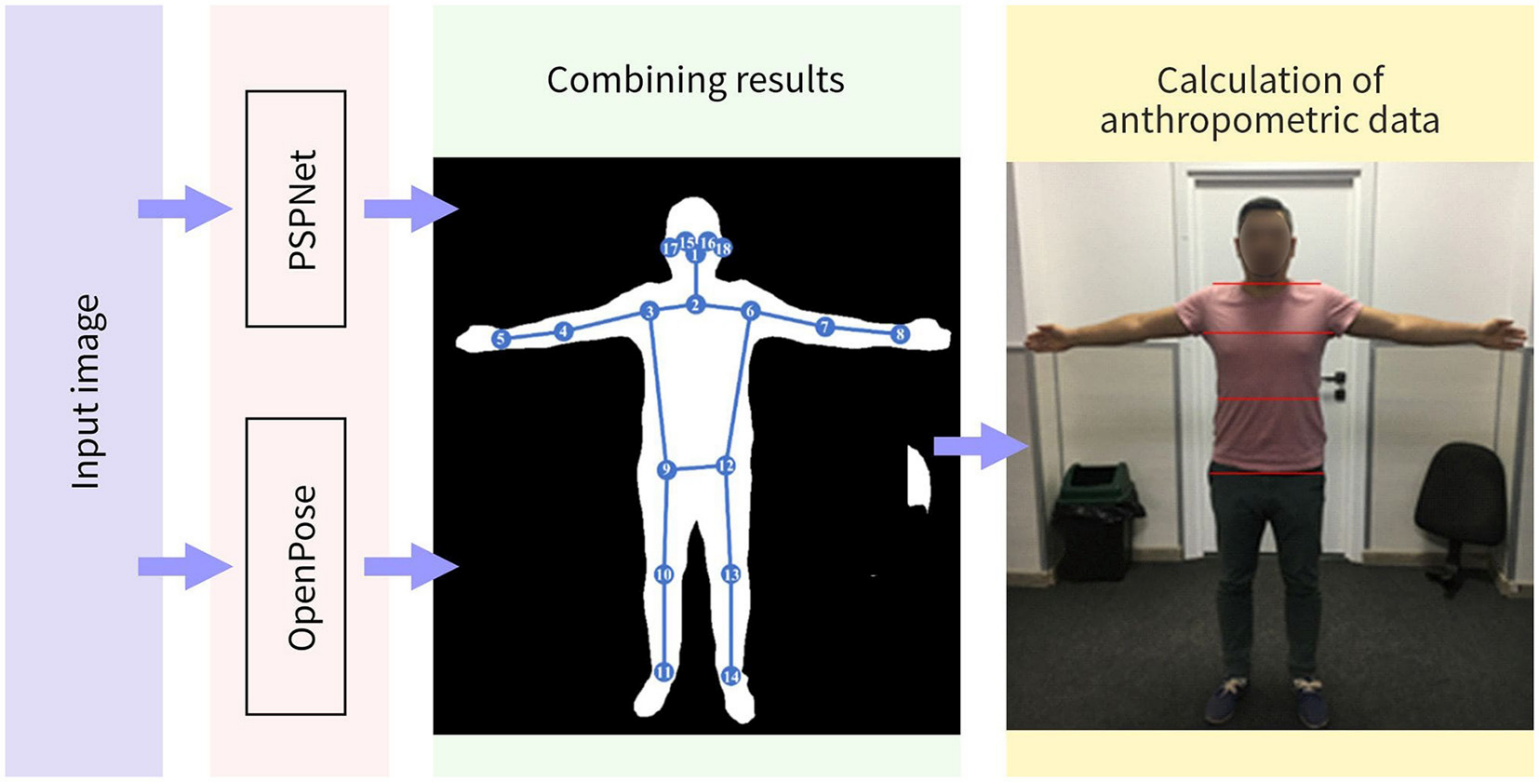
The process of combining the results.

Before delving into the description of the semantic segmentation method itself, an approach to acquiring anthropometric data from the image of body shape should be described. A method of deriving anthropometric measurements from an image of the body based on a combination of two pre-trained models was previously developed (Absadyk and Turar, 2020). The first model is a semantic segmentation model based on PSPNet (Zhao et al., 2016), and the second is OpenPose model (Cao et al., 2017) that is used to obtain key points of the joints and face. In this approach, the images of people wearing skinny and tight clothes are necessary that do not distort anthropometric parameters. The scheme of combination of models is presented in Figure 1.

Algorithm for determining the characteristic points from combined data:

1. Leg length: determined by calculating the average length of the segments *P*_9_*P*_11_ and *P*_12_*P*_14_.

$$\begin{array}{l} S_{leg}=\frac{\sqrt{{\left(x_{12}-x_{14}\right)}^{2}+{\left(y_{12}-y_{14}\right)}^{2}}}{2}\\ \;+\frac{\sqrt{{\left(x_{9}-x_{11}\right)}^{2}+{\left(y_{9}-y_{11}\right)}^{2}}}{2}; \end{array}$$

2. Thallium length: determined by calculating the smallest distance of the body's extreme points at the thallium's level. The thallium level is located at the intersection of segments *P*_3_*P*_12_ and *P*_6_*P*_9_

$$\begin{array}{l} S_{tallium}={min}_{x}\left(x_{c},\;y_{c},0\right)-{min}_{x}\left(x_{c},\;y_{c},180\right); \end{array}$$

where (*x*_*c*_, *y*_*c*_) are the coordinates of the intersection point of the segments, *P*_3_*P*_12_ and *P*_6_*P*_9_, and the third argument is the direction in which the function *min*_*x*_ finds the X coordinates of the closest point of the object's contour.

3. Arm length: determined by calculating the distance between the points of intersection of the contour of the body with a straight line released from 3 and 6 points at a 135 and 45 degree angle, respectively.

$$\begin{array}{l} S_{shoulder}=\;{min}_{x}\left(x_{6},\;y_{6},45\right)-{min}_{x}\left(x_{3},\;y_{3},135\right); \end{array}$$

4. Hip length: determined by calculating the distance of the extreme points of the body at the level of 12 and 9 points.

$$\begin{array}{l} S_{Hip}=\;{min}_{x}\left(x_{12},\;y_{12},0\right)-{min}_{x}\left(x_{9},\;y_{9},180\right); \end{array}$$

5. Neck length: determined by calculating the smallest distance of the extreme points of the body along a straight neck *P*_2_*P*_1_.

$$\begin{array}{l} S_{neck}=\;S_{min}\left(P_{2}P_{1}\right); \end{array}$$

6. Chest-level point spacing: First, the closest points to points 3 and 6 in the area *P*_4_*P*_3_*P*_9_ and *P*_7_*P*_6_*P*_12_ accordingly are determined, then the distance between these points is calculated.

$$\begin{array}{l} S_{chest}=\;S_{min}\left(P_{6}P_{7},P_{6}P_{12}\right)-S_{min}\left(P_{3}P_{4},P_{3}P_{9}\right); \end{array}$$

7. Arm length: determined by calculating the length of the segments *P*_3_*P*_5_ and *P*_6_*P*_8_, then find their average value.

$$\begin{array}{l} S_{arm}=\frac{\left(S\left(P_{6}P_{8}\right)+S\left(P_{3}P_{5}\right)\right)}{2}; \end{array}$$

Thus, seven significant measurements of the human body have been identified. However, it is important to note that the true positions of the key points may not align with the perimeter of the clothing worn by the individual.

Further, the construction of a semantic segmentation model that minimizes the error of body shape determination based on the corresponding metric is described. Proper construction of such a model first requires the creation of a specifically labeled dataset. In this case, a synthetic dataset must be created. The most fitting model of semantic segmentation is then selected by retraining various models on the dataset and providing further comparisons.

### 4.1 Development of synthetic data

Utilizing synthetic data offers a significant benefit in that it allows for precise control over the characteristics of objects and scenes used in training the model. The NVIDIA Omniverse Replicator offers tools to introduce variability by randomizing objects within a scene. Notably, this technology employs ray tracing for rendering, enabling the generation of highly realistic data. The NVIDIA Omniverse Replicator comprises a flexible set of APIs that facilitate the creation of physically accurate synthetic data to train computer vision networks. This empowers deep learning engineers and researchers to expedite model training, enhance performance, and explore new possibilities in model development, thus overcoming limitations posed by dataset availability and annotations (NVidia, 2018). In the synthetic dataset, as in any image segmentation dataset, the input is a realistic image of a human, and the output is an image with a mask of the area of interest.

Input requirements:

- Input image orientation: Images in vertical orientation. As of the third quarter of 2022 around 91 percent reported accessed the internet via smartphones (Petrosyan, 2020). Therefore, in this work, images taken on a phone were also focused on, increasing the possibility of using the algorithm.
- Objects in the image: The main object in the image should be a person, who should be positioned completely within the image and in the center. No objects should overlap the person; objects can be placed behind or near the person. Only one person should be present in the image. The background can contain anything except other people.
- Surroundings: Images can be taken inside or outside the building.
- Posture: the person stands directly in front of the camera, arms spread apart, legs slightly wider than shoulder level, head looking forward at the camera.

Output requirements:

- In the output image, only the area of person body should be outlined by masking pixels. Each pixel belongs only to class of human (1) or background (0).

To utilize the Replicator, it is necessary to collect three-dimensional models of all objects involved in creating synthetic data. In this study, realistic three-dimensional models of seven distinct body types, each with varying body parameters, were generated using the open-source Blender software.

A different combination of outerwear, trousers and shoes was created for different body types. All sorts of furniture and trees were selected, and walls with different numbers of windows were modeled. The floor and ceiling were left flat. Pictures were marked on the wall behind the man. All models shown in Table 1.

**Table 1:** 3D models parameters.

**3D models**	**Number of elements**	**Types**	**Randomization functions** ^a^	**Examples**
Body	7	From thin to fat	rep.randomizer.scatter_2d(body_scatter) rep.modify.pose(rotation)	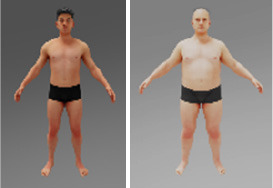
Outerwear	17	Shirt, sweater, t-shirt, etc.	rep.create.material_omnipbr(cloth_texture) custom_randomizer_function()	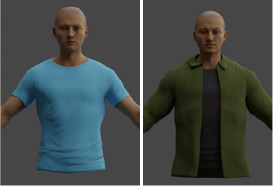
Pants	6	Jeans, chinos, shorts, etc.	rep.create.material_omnipbr(cloth _texture) custom_randomizer_function()	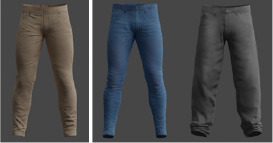
Shoes	8	Boots, shoes, sneakers, etc.	rep.create.material_omnipbr(cloth _texture) custom_randomizer_function()	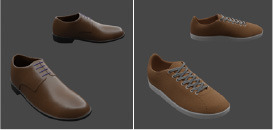
Furniture	28	Sofa, table, chairs, etc.	rep.randomizer.scatter_2d(furniture_scatter) modify.pose(rotation)	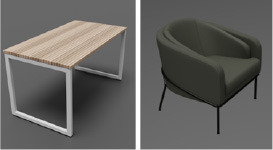
Walls	4	With 1, 2, 3 windows and without window	custom_randomizer_function() rep.create.material_omnipbr(wall_texture) rep.modify.pose(rotation)	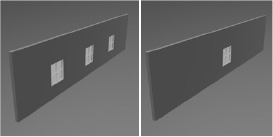
Trees	15	Conifers, pines, bushes, etc.	rep.randomizer.scatter_2d(tree_scatter) modify.pose(rotation)	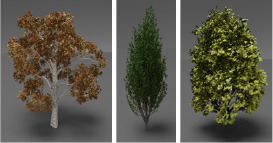
Floor and ceiling	1	Flat floor and ceiling	rep.create.material_omnipbr(floor_texture) custom_randomizer_function()	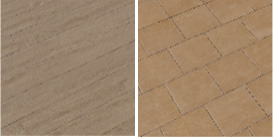
Frames	5	Different pictures in a frame	rep.randomizer.scatter_2d(frame_scatter)	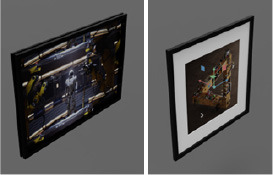
Lighting	5	Regular lights	rep.create.light(temperature, intensity, scale) rep.randomizer.scatter_2d(light_scatter)	

^a^omni.replicator.core has been imported as rep.

For the texture of clothes, floors, and walls from open sources, images with different textures were collected. HDRI environments for backgrounds have been imported from Omniverse's open library of 3d assets. The number of texture images is shown in Table 2.

**Table 2:** The number of texture images.

**Textures**	**Number of elements**	**Types**	**Examples**
Cloth	52	Denim, checkered, knitted, polished, etc.	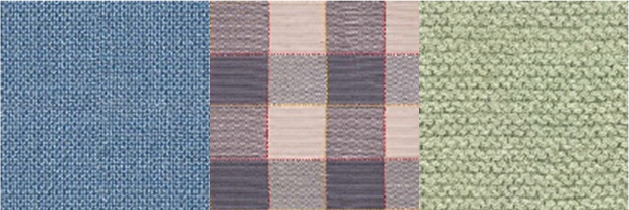
Floor	64	Parquet, carpet, tiles, etc.	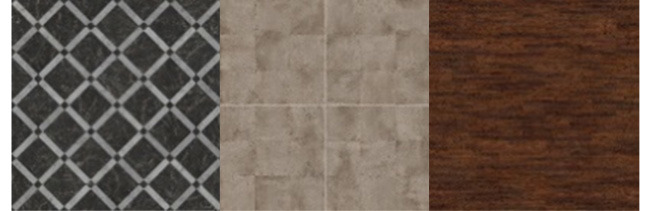
Walls	49	Brick, tiles, concrete, etc.	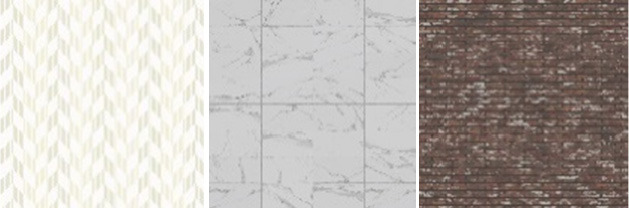
Environment	10	HDRI images of clear, cloudy day, night, etc.	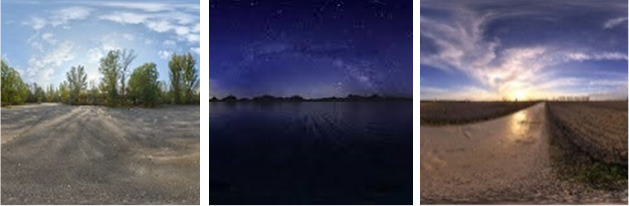

**Figure 2:**
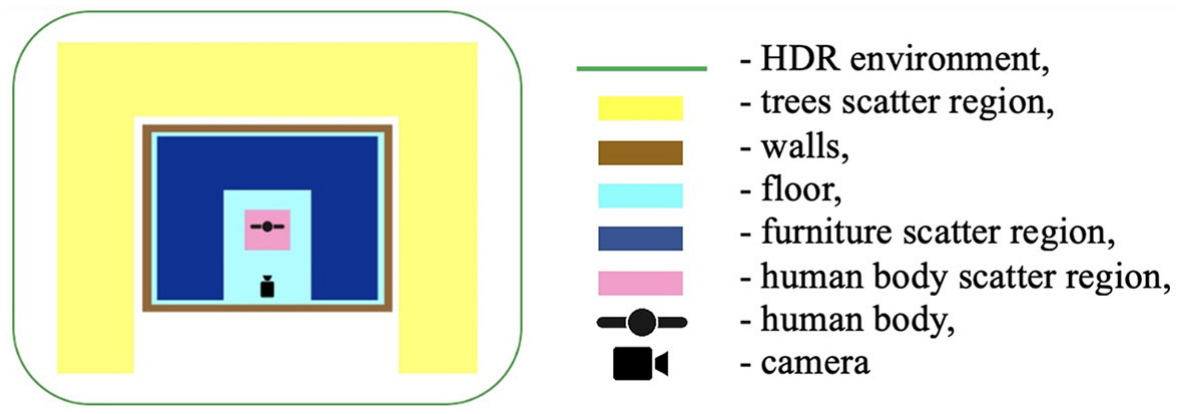
3D objects placement setup.

All objects were placed according to the following scheme below (Figure 2).

Object placement: randomization functions were used to place objects such as furniture, trees, and frames within the scene. These objects were scattered using the rep.randomizer.scatter_2d function, ensuring they did not overlap with the human subject.

Lighting conditions: the lighting conditions were varied by changing the position, intensity, and temperature of the lights. This was done using the rep.create.light function, with parameters set to introduce realistic shadows and highlights.

Texture variability: different textures were applied to the objects and backgrounds using the rep.create.material_omnipbr function. A wide range of textures was included, from denim and checkered fabrics for clothing to parquet and tiled surfaces for floors.

Body types: the dataset included seven distinct body types, modeled in Blender, ranging from thin to obese. The body types were assigned random combinations of outerwear, trousers, and shoes to simulate a variety of real-world clothing scenarios.

Camera setup: the camera's position and focal length were randomized within specified bounds to simulate different perspectives. Focal lengths of 24 mm, 30 mm, and 35 mm were used, with distances from the camera to the subject adjusted accordingly to maintain consistent framing.

Image generation: a randomizer specifically for human clothing was developed, including several types of shoes, trousers, and clothes. The randomizer's function was to generate different combinations of these items. The generation of 500 images required approximately 12 hours on our device. By utilizing random.seed, the Replicator was able to repeat the same combination of random sets of objects for a single seed. The Replicator was launched twice with the same seed: the first time considering all objects and clothes, and the second time isolating only the human body while hiding all other objects for binary mask. The computational experiments reported in this paper were conducted on a high-performance workstation with the following specifications:

- CPU: Intel Core i9-10900K @ 3.7GHz
- RAM: 128GB DDR4 @ 3200MHz
- 2TB NVMe PCIe M.2 Solid State Drive
- GPU: NVIDIA GeForce RTX 3090 with 24 GB of GDDR6X VRAM

As a result, an input image with all random objects and clothes with random textures was obtained, along with a silhouette of a person's body without considering the clothes. A total of 7,254 images of a person in different clothes, posture and surroundings were generated, and 7,254 images with semantic segmentation of the human body, respectively. The size of the image is 600 px wide and 900 px high. Seven different physiques. The number of pictures for each of them is shown in Table 3. Image augmentation technology was used to increase the amount of data. All images have been flipped horizontally and rotated. As a result, the number of images was more than 22,000.

**Table 3:** Number of pictures for each body type.

**Body types**	** 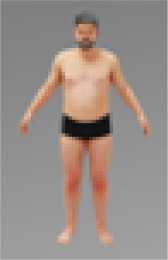 **	** 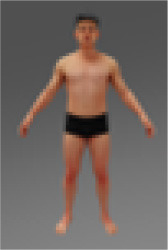 **	** 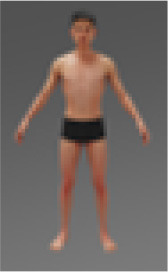 **	** 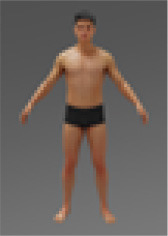 **	** 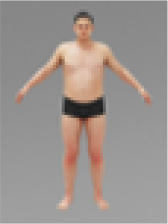 **	** 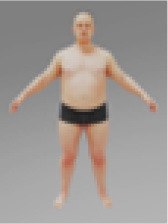 **	** 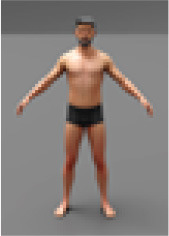 **
Number of images	1,000	1,272	1,000	595	1,000	1,250	1,137
Height (cm)	183	176	173	175	179	181	185

### 4.2 Modified CNN models

#### 4.2.1 Backbones

Some of the popular CNN architectures, such as ResNet50 and ResNet101, have been utilized. A residual network that consists of 50 and 101 layers respectively and uses skip connections to bypass some layers and avoid the problem of vanishing gradients (He et al., 2016), EfficientNet: A family of neural networks that are designed to be efficient and scalable (Tan and Le, 2019). and VGG19: A very deep convolutional network that consists of 19 layers and uses small filters (3x3) and max-pooling layers to reduce the spatial dimensions of the feature maps (Sudha and Ganeshbabu, 2020) as a backbone. They have been trained on ImageNet dataset.

#### 4.2.2 Semantic segmentation models

Based on the input data and the task at hand, some CNN architectures were considered. The selection of specific architectures such as U-Net, SegNet, DeepLabV3, and PSPNet was driven by their demonstrated effectiveness in semantic segmentation tasks. U-Net was selected for its symmetric encoder-decoder architecture with skip connections, which facilitates precise localization by combining high-resolution features from the encoder with upsampled outputs (Ronneberger et al., 2015). SegNet was chosen for its efficient use of encoder feature maps to perform nonlinear upsampling in the decoder, making it particularly effective for pixel-wise classification tasks (Badrinarayanan et al., 2017). DeepLabV3 was selected due to its use of atrous convolution, which allows for larger receptive fields without loss of resolution, and its spatial pyramid pooling that captures multi-scale contextual information (Chen et al., 2018). PSPNet was included for its pyramid pooling module that effectively captures global context information at different scales (Zhao et al., 2016).

The modifications to architectures were primarily driven by the need to enhance accuracy. Specifically, the resolution of the output layer was increased to produce a higher-resolution binary mask, which is critical for accurately extracting anthropometric data. This increase in resolution ensures that the finer details of the human body segmentation are captured more precisely, thereby improving the accuracy of the anthropometric measurements derived from these masks. The modifications involved incorporating additional convolutional layers and bilinear interpolation techniques in the upsampling process, which enhanced the spatial resolution of the segmentation maps and led to more detailed and accurate segmentation outputs.

##### 4.2.2.1 Modified U-Net

U-Net was developed for biomedical image segmentation, we can use it for binary semantic segmentation tasks. The architecture stems from the FCN (Roman, 2023). The main idea is to supplement a usual contracting network by successive layers, where pooling operations are replaced by upsampling operators.

**Figure 3:**
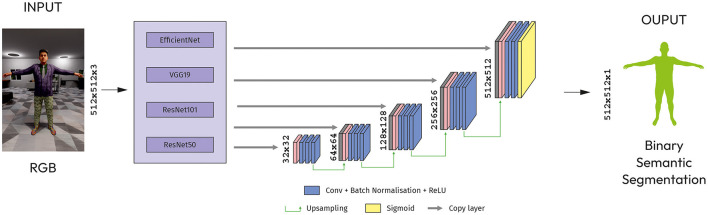
Modified U-Net with CNN models as an encoder.

The architecture has five blocks of up-convolutional layers, each followed by a ReLU activation function. The up-convolutional layers increase the spatial resolution of the feature maps by a factor of two. Last block has a main input layer (512x152x3) for concatenation with upsampled layer by 2x2 from output of previous block (Figure 3).

##### 4.2.2.2 Modified SegNet

**Figure 4:**
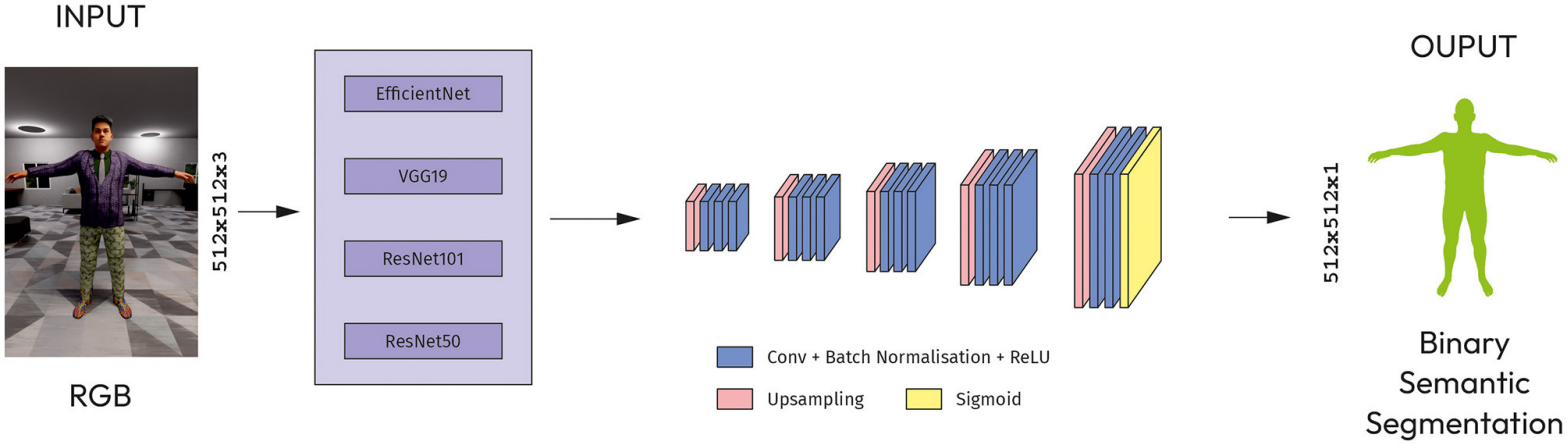
Modified SegNet with CNN models as an encoder.

SegNet is a trainable segmentation architecture that consists of an encoder network, a corresponding decoder network followed by a pixel-wise classification layer (Badrinarayanan et al., 2017). The architecture has five blocks of up-convolutional layers, each followed by a ReLU activation function (Figure 4).

##### 4.2.2.3 Modified DeepLabV3

The DeepLabV3 model has the following architecture: Features are extracted from the backbone network (VGG, EfficientNet, ResNet). To control the size of the feature map, atrous convolution is used in the last few blocks of the backbone. Apart from using Atrous Convolution, DeepLabV3 uses an improved ASPP module by including batch normalization and image-level features (Chen et al., 2018).

**Figure 5:**
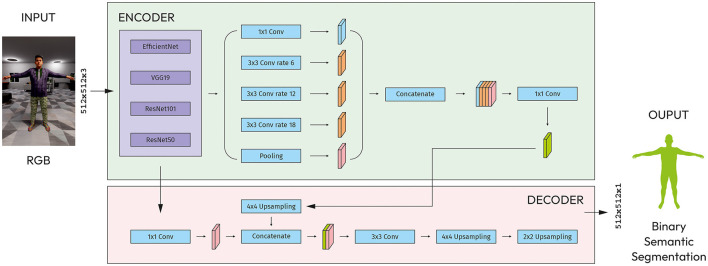
Modified DeepLabV3 with CNN models as an encoder.

The ASPP module is a set of parallel dilated convolutions with 6, 12, 18 dilation rates. The output of each convolutional layer is then concatenated along the channel dimension to create a multi-scale feature representation. The upsampled by 4x4 feature map is concatenated with the feature map output by the encoder stage via a skip connection. 2 Upsampling layers with sizes 4x4 and 2x2 followed by 1x1 convolutional layer and then Sigmoid activation function (Figure 5).

##### 4.2.2.4 Modified PSPNet

The PSPNet encoder contains the CNN backbone with dilated convolutions along with the pyramid pooling module. The output of the backbone is fed into a pyramid pooling module, which captures contextual information at multiple scales. The pyramid pooling module consists of four parallel pooling layers, each with a (1, 1), (2, 2), (4, 4), (8, 8) bin size.

**Figure 6:**
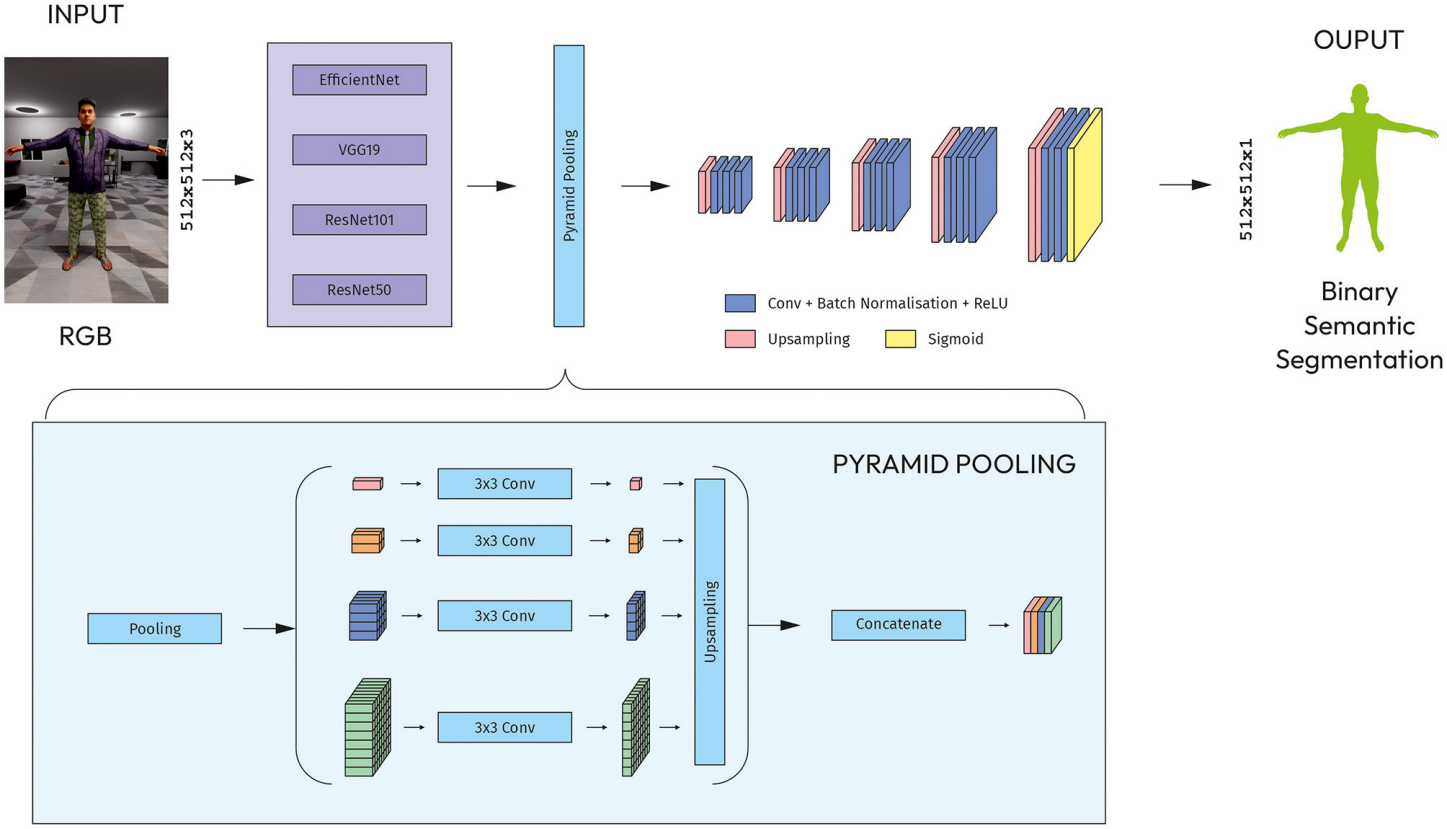
Modified PSPNet with CNN models as an encoder.

The output of the pyramid pooling module goes through five blocks of up-convolutional layers that uses bilinear interpolation. The feature map from last block is processed by a series of convolutional layers to reduce the number of channels and output a feature map with one channel as the number of classes to be segmented (Figure 6).

## 5 Results and analysis

### 5.1 Quantitative results

The parameters for training were as follows: Adam optimizer with *learning*_*rate* = 0.001, the loss function is binary-crossentropy that is typical for binary segmentation tasks. The dataset was split into two parts using *validation*_*split* = 0.2. To avoid overfitting, we set *patience* = 5, *monitor* =′*val*_*loss*′, *epochs* = 50, *batch*_*size* = 8 (Table 4).

**Table 4:** CNN models with the best results.

**Parameters**	**U-Net with EfficientNet**	**SegNet with EfficientNet**	**DeepLabV3 with VGG19**	**PSPNet with VGG19**
Total parameters	9,201,465	7,454,805	21,486,337	29,198,257
Training accuracy	0.998296	0.996924	0.998348	0.997840
Training loss	0.003964	0.007163	0.003772	0.004974
Validation accuracy	0.997578	0.996091	0.997496	0.996516
Validation loss	0.006075	0.009327	0.006598	0.009416
Precision	0.991013	0.973065	0.982789	0.986469
Recall	0.986196	0.986311	0.974841	0.982710
IoU	0.977454	0.960099	0.958478	0.969640

For training, all models were run with the same parameters. The highest training accuracy was shown by the U-Net model with the EfficientNet and DeepLabV3 with the VGG19 backbones (Table 4). The U-Net with EffisientNet shows less accuracy in training than DeepLabV3 with VGG19, but the total number of parameters in DeepLabV3 with VGG19 is more than two times. Despite this, the difference in accuracy in training is minimal. When validating, U-Net with EfficeintNet shows greater accuracy than the second model. For all other indicators, the situation is the same. The U-Net with EfficientNet as a backbone model is ahead of all other models in Precision, Recall, and IoU.

### 5.2 Visual comparisons

**Figure 7:**
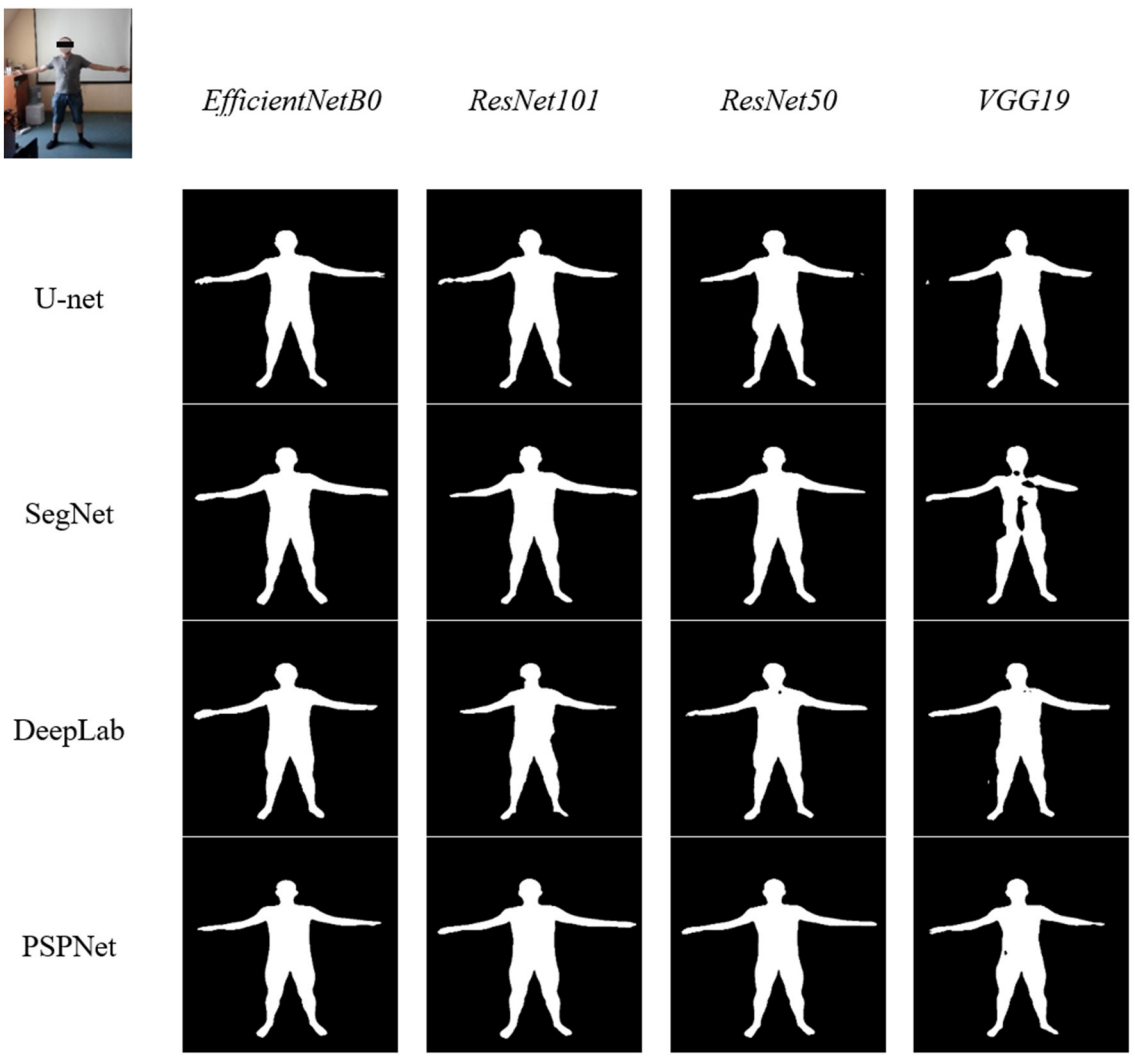
Different model tests on real images.

All trained models were tested on real data. Surprisingly, all DeepLabV3 models showed the worst results of all the others. U-Net and SegNet with EfficientNet as a backbone and all PSPNet models except the ones with VGG19 as a backbone have good results (Figure 7).

The visual comparison of the segmentation results reveals key differences between the models. U-Net model produces highly accurate boundaries, closely matching the true segmentation, while others often appear to under-segment the images, missing finer details. PSPNet with EfficientNet, on the other hand, shows a tendency to over-segment certain regions, especially in the legs and body regions. Despite these differences, all five models perform well in terms of the general quality of segmentation. Overall, while each model has its strengths and weaknesses, U-Net and SegNet with EfficientNet provide the most accurate and consistent results across a variety of real images, even though SegNet models showed some of the worst results in validation training.

### 5.3 Robustness and generalization

**Figure 8:**
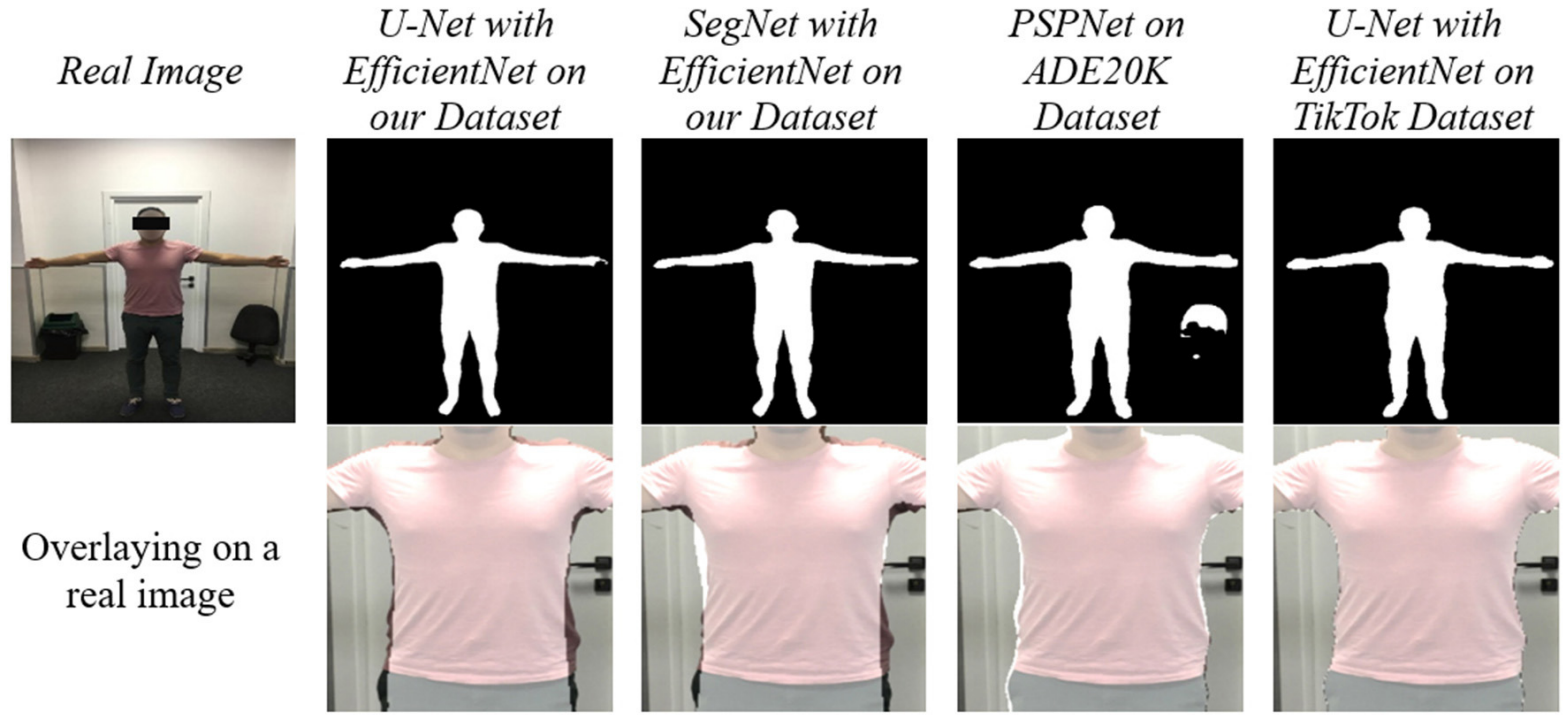
Models comparison.

For comparison, the models with the best results on the TikTok Dataset were trained, along with the previously used and initially pre-trained PSPNet. All the resulting models were tested on real images. As expected, the models trained on our dataset performed better in terms of determining a person's true physique. At the same time, the rest defined the silhouette of a person with clothes. PSPNet also correctly detects the silhouette of a person with clothes; the results are not very solid, and there are errors in some areas. The results of SegNet with EfficientNet are solid and cover more than required. This can be seen when overlaying on a real image (Figure 8).

Results of SegNet and UNet models were compared with EfficientNet as an encoder trained using IoU loss and Binary-crossentropy loss (Table 5).

**Table 5:** UNet and SegNet models with EfficientNet as a backbone trained using different loss functions.

**Parameters**	**IoU loss used**		**Binary-crossentropy loss used**	
	**U-Net with EfficientNet**	**SegNet with EfficientNet**	**U-Net with EfficientNet**	**SegNet with EfficientNet**
Accuracy	0.998740	0.997973	0.998404	0.997124
Precision	0.980982	0.991212	0.991013	0.973065
Recall	0.990318	0.990829	0.986196	0.986311
IoU	0.971663	0.982201	0.977454	0.960099
IoU loss	0.017799	0.028337	0.022545	0.039901
Binary-crossentropy loss	0.018111	0.028633	0.003837	0.006881

U-Net models with EfficientNet exhibit better accuracy results, with their values being nearly identical. The primary distinctions lie in the losses incurred. For models employing an IoU loss, the loss values are nearly equivalent, whereas for those utilizing a Binary-crossentropy loss, the loss rates are considerably lower compared to IoU loss. The resultant model was tested on real images featuring a man wearing a sweatshirt. The tests demonstrated that the model takes clothing into account when determining the shape of a human body.

**Figure 9:**
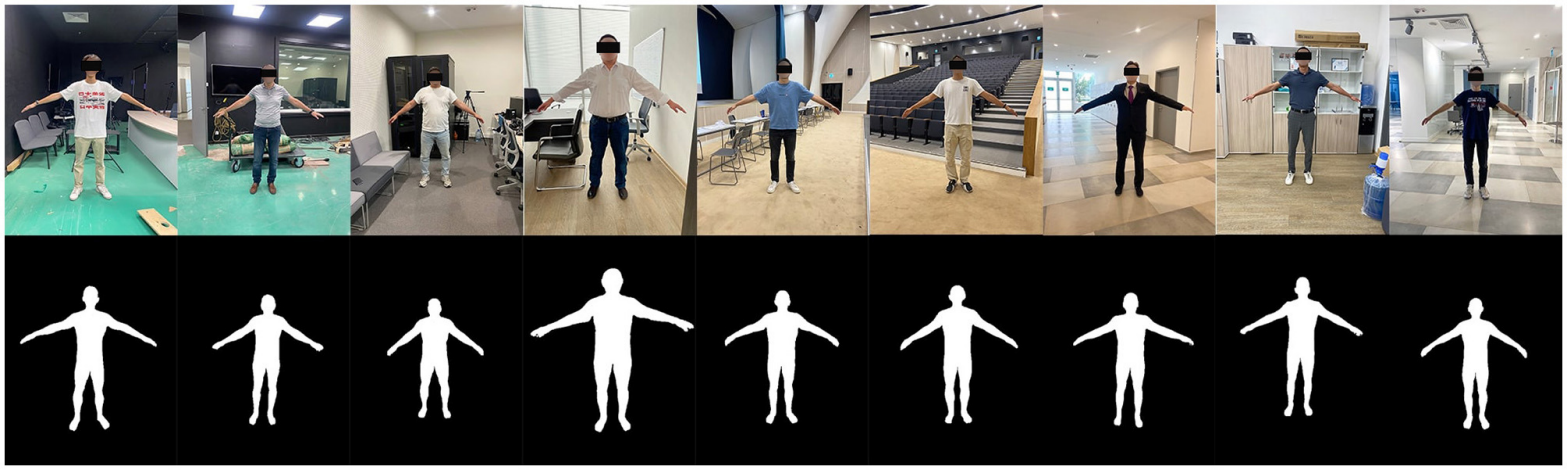
Results of testing our final model on real human subjects.

Extensive testing was conducted on the model using real human images featuring a variety of clothing types and body shapes to evaluate the robustness and accuracy of the segmentation and anthropometric data extraction algorithms of this study. Specifically, testing was performed on real human subjects, ensuring diverse representations in terms of clothing overlays and body types (Figure 9). The performance of the algorithm was then compared with actual anthropometric measurements. This comparative analysis involved 9 participants, who provided their real anthropometric data for validation purposes. To calculate the measurements, we have used the algorithm for determining characteristic points from combined data. The detailed results of this analysis, including the comparative performance metrics, are presented in the Table 6.

**Table 6:** Deviation of algorithm for determining characteristic points from real data (cm).

**Person**	**Neck**	**Shoulder**	**Chest**	**Thallium**	**Hip**
Person 1	1.54	0.19	4.35	3.82	3.39
Person 2	0.64	-1.4	0.52	3.61	-0.71
Person 3	-2.36	1.3	3.11	4.4	4.43
Person 4	0.17	-2.73	-1.52	-2.58	-1.94
Person 5	-0.51	-2.62	2.35	2.82	4.44
Person 6	-0.94	2.41	3.5	-1.55	4.29
Person 7	-0.35	1.1	-2.15	-5.76	-3.98
Person 8	-0.27	3.02	-1.51	-3.38	-4.29
Person 9	-0.04	-0.34	1.75	-3.42	-4.03
Average	-0.235	-0.1	1.15	-0.22	0.17

## 6 Conclusion

Various artificial intelligence technologies are used to obtain anthropometric data of the body without contact with the body. However, many of them have limitations or are tailored to specific conditions, which reduces the feasibility of utilizing these technologies. Existing datasets are designed to obtain the silhouette of a person with clothes, which cannot be applied in task. In this article, a method for creating a dataset to determine the true silhouette of a person, independent of clothing, is proposed. The resulting dataset is then applied to several popular architectures.

**Figure 10:**
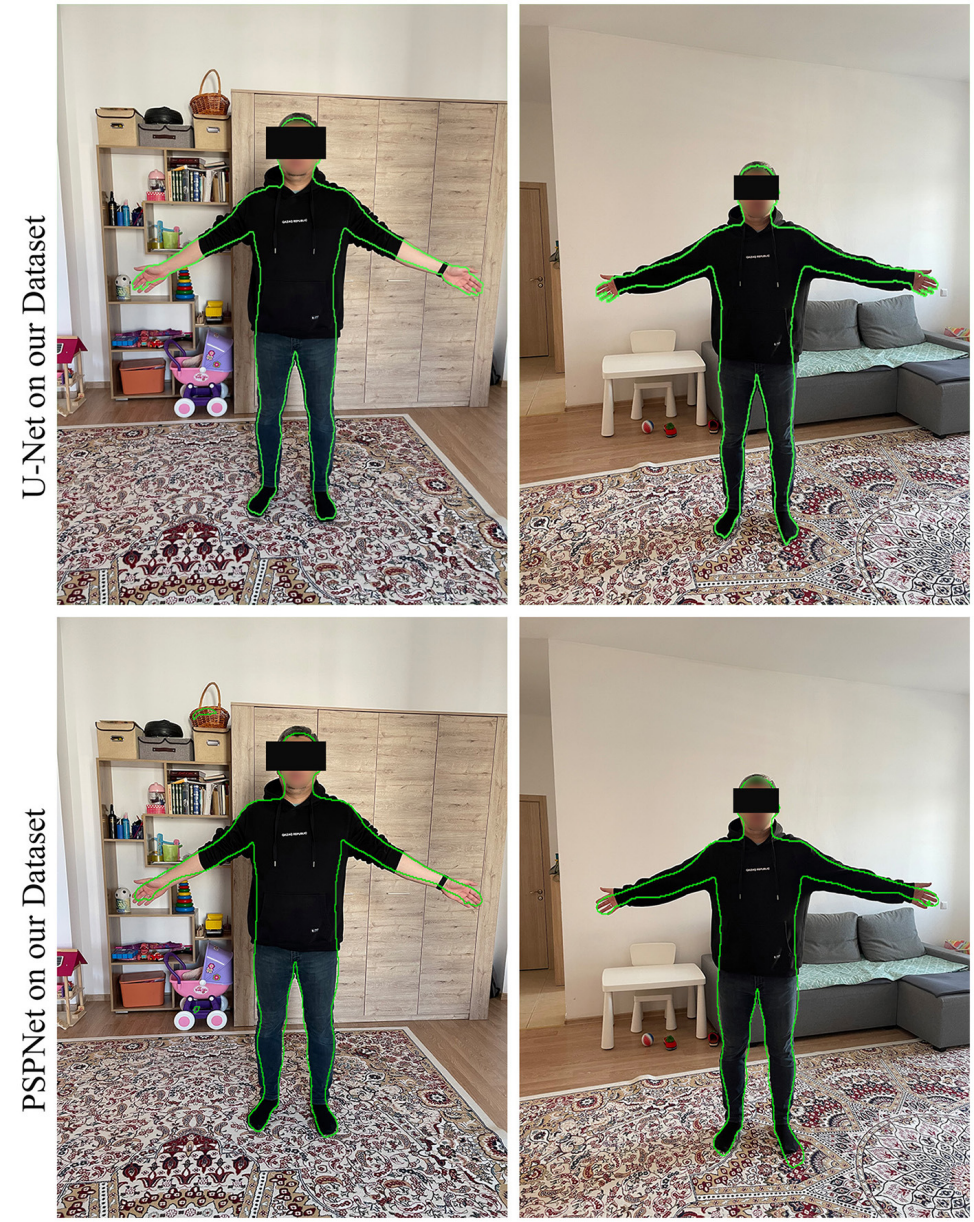
Results of testing our final model (UNet with EfficientNet) with PSPNet trained on our dataset.

As a result of the work performed, a training dataset of more than 22000 pictures with semantic segmentation of the human body, taking into account clothing, was obtained. The dataset were used to train popular neural networks of semantic segmentation with CNN architectures as a backbone. The trained modules are tested with real data. The accuracy of the data allows the dataset to be used to determine a person's true body. Among the models created, the best result was shown by U-Net with the EfficientNet basis. Testing on real data showed good results. To summarize the above, dataset provided in this study can be used to derive human anthropometric data from real images (Figure 10).

When evaluating the accuracy of the anthropometric data extraction algorithm, performance was analyzed across various body measurements, including Neck, Shoulder, Chest, Thallium, and Hip. The dataset highlights both the strengths and areas for improvement of the algorithm. The algorithm demonstrates relatively consistent accuracy in extracting Neck and Shoulder measurements, indicated by their lower standard deviations and means close to zero. This suggests a balanced extraction process without significant bias. However, the Chest measurements, despite having a positive mean, exhibit higher variability, indicating the need for better accuracy and consistency.

Notably, Thallium and Hip measurements present the greatest challenge, showing the highest standard deviations and widest ranges. This variability points to substantial inconsistencies in the data extraction process, underscoring the need for refinement in these areas. To improve the algorithm's performance, we will focusing on enhancing the extraction methods for Thallium and Hip measurements, conducting thorough error analysis to address the causes of high variability, and increasing the diversity of test cases to ensure robust performance across different scenarios. Through the implementation of these improvements, the aim is to achieve more precise and consistent measurements, thereby enhancing the overall reliability and applicability of the algorithm.

The practical implications of this study are significant for the e-commerce and fashion industries. Accurate anthropometric data extraction can revolutionize virtual fitting rooms, allowing customers to try on clothes virtually and obtain better fitting garments, thereby reducing return rates and associated logistical costs.

A limitation of this work is that while the dataset created assists in determining the size of a person's body, it necessitates combining the results of the resulting model with the model for determining the person's posture, significantly slowing down the result acquisition process. To address this issue, the next phase of the work will explore the development of a new dataset for identifying anthropometric keypoints from photography. This set will then be tested on existing CNN architectures for landmark detection.

## Data availability statement

The datasets presented in this study can be found in online repositories. The names of the repository/repositories and accession number(s) can be found below: https://www.kaggle.com/datasets/azatabsadyk/synthropo-front.

## Ethics statement

Written informed consent was obtained from the individuals for the publication of any potentially identifiable images or data included in this article.

## Author contributions

AA: Data curation, Formal analysis, Funding acquisition, Investigation, Methodology, Resources, Software, Visualization, Writing – original draft. OT: Supervision, Writing – review & editing. DA-Z: Conceptualization, Project administration, Supervision, Writing – review & editing.
